# Sexual Dimorphism in Energy Metabolism of Wistar Rats Using Data Analysis

**DOI:** 10.3390/molecules25102353

**Published:** 2020-05-18

**Authors:** Andrea Leskanicova, Olga Chovancova, Marian Babincak, Ludmila Verboova, Zuzana Benetinova, Denisa Macekova, Jozef Kostolny, Benadik Smajda, Terezia Kiskova

**Affiliations:** 1 Institute of Biology and Ecology, Faculty of Sciences, University of Pavol Jozef Šafárik in Košice, Šrobárova 2, 041 80 Košice, Slovakia; andrea.stafurikova@student.upjs.sk (A.L.); marian.babincak@student.upjs.sk (M.B.); 2 Department of Informatics, Faculty of Management Sciences and Informatics, University of Žilina, Univerzitná 8215/1, 010 26 Žilina, Slovakia; olga.chovancova@fri.uniza.sk (O.C.); denisa.macekova@fri.uniza.sk (D.M.); jozef.kostolny@fri.uniza.sk (J.K.); 3 Department of Pathology, Faculty of Medicine, University of Pavol Jozef Šafárik in Košice, Rastislavova 43, 040 01 Košice, Slovakia; ludmila.verboova@upjs.sk (L.V.); zuzana.benetinova@upjs.sk (Z.B.)

**Keywords:** metabolomics, FIA-MS/MS and LC-MS/MS, energy metabolism, sexual dimorphism, acylcarnitines, amino acids

## Abstract

The prevalence of some chronic diseases, such as cancer or neurodegenerative disorders, differs between sexes. Animal models provide an important tool to adopt potential therapies from preclinical studies to humans. Laboratory rats are the most popular animals in toxicology, neurobehavioral, or cancer research. Our study aimed to reveal the basic differences in blood metabolome (amino acids, biogenic amines, and acylcarnitines) of the adult male (*n* = 10) and female (*n* = 10) Wistar rats. Partial least square-discrimination analysis (PLS-DA) and a variance im portance in projection (VIP) score was used to identify the key sex-specific metabolites. All groups of metabolites, as the main markers of energy metabolism, showed a significant sex-dependent pattern. The most important features calculated in PLS-DA according to VIP score were free carnitine (C0), tyrosine (Tyr), and acylcarnitine C5-OH. While aromatic amino acids, such as Tyr and phenylalanine (Phe), were significantly elevated in the blood plasma of males, tryptophan (Trp) was found in higher levels in the blood plasma of females. Besides, significant sex-related changes in urea cycle were found. Our study provides an important insight into sex-specific differences in energy metabolism in rats and indicates that further studies should consider sex as the main aspect in design and data interpretation.

## 1. Introduction

Many studies, including brain research, evolutionary psychology, and anthropology, demonstrate that males and females are physically and mentally different. Sex differences are prominent also in many disorders, such as mood and anxiety disorders [1], cancer [2], cardiovascular disorders [3,4], and diabetes mellitus type 2 as well as chronic kidney diseases and renal dysfunctions [5,6,7,8]. Because plenty of sex differences are primarily in the structure, function, and stress responsivity of the brain, as well as the differences in exposure to reproductive hormones, social expectations, and experiences, the challenge is to understand which sex differences are relevant to affect illnesses [9].

Animal models provide a key tool to adopt potential therapies from preclinical studies to humans [10]. Notably, animal research provides a degree of experimental control and precision not usually feasible in studies using human subjects [11]. By the late 18th or early 19th century, rats became the most commonly used experimental animals in numerous biomedical research, as they have been recognized as the preeminent model mammalian system [12]. However, the rats used in most experiments are males. Researchers usually avoid using females because of their reproductive cycles and hormone fluctuations that may confound the results of their studies [13,14]. But, the susceptibility to various diseases as well as to their treatment varies considerably among males and females.

Understanding the basic metabolism in males and females is the first step in revealing further differences between sexes. Aside from ovarian cyclicity and menopause, there are many factors (e.g., body composition, regional fat distribution, aerobic fitness, and so on, all of which are known to affect sex-specific substrate metabolism) that might complicate the interpretation of the results obtained from females [15]. Exercising males, compared with females, have a greater increase in leucine oxidation but not lysine levels. This indicates that exercising males increase their need for amino acids to fuel energy needs, whereas females increase their mobilization of fat, thereby requiring less alternative fuels such as carbohydrate and amino acids [16]. Substrate availability during exercise appears to modulate the amino acid oxidation differences between sexes [17,18,19]. The aim of our study was to reveal the main variances in energy metabolism using liquid chromatography-tandem mass spectrometry-based targeted metabolomics’ measurements in the blood plasma of healthy male and female Wistar rats.

## 2. Results

**Figure 1:**
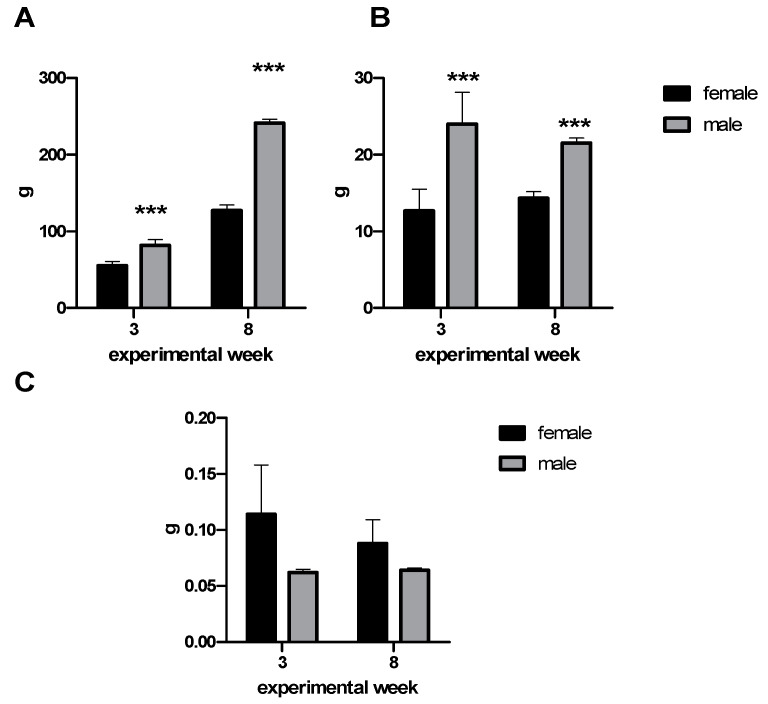
(**A**) Body mass gain, (**B**) food intake, and (**C**) food intake per gram of body weight during the third and eighth experimental weeks. Data are expressed as mean ± SD. Significance is given by *** *p* < 0.001.

During the experiment, the body mass gain was evaluated. Male rats had significantly higher body mass gain (*p* < 0.001) throughout the experiment. In the third and eighth experimental weeks, food intake was monitored. Males had significantly elevated food intake (*p* < 0.001). However, there were no differences between male and female groups in food intake per gram of body weight (Figure 1).

**Figure 2:**
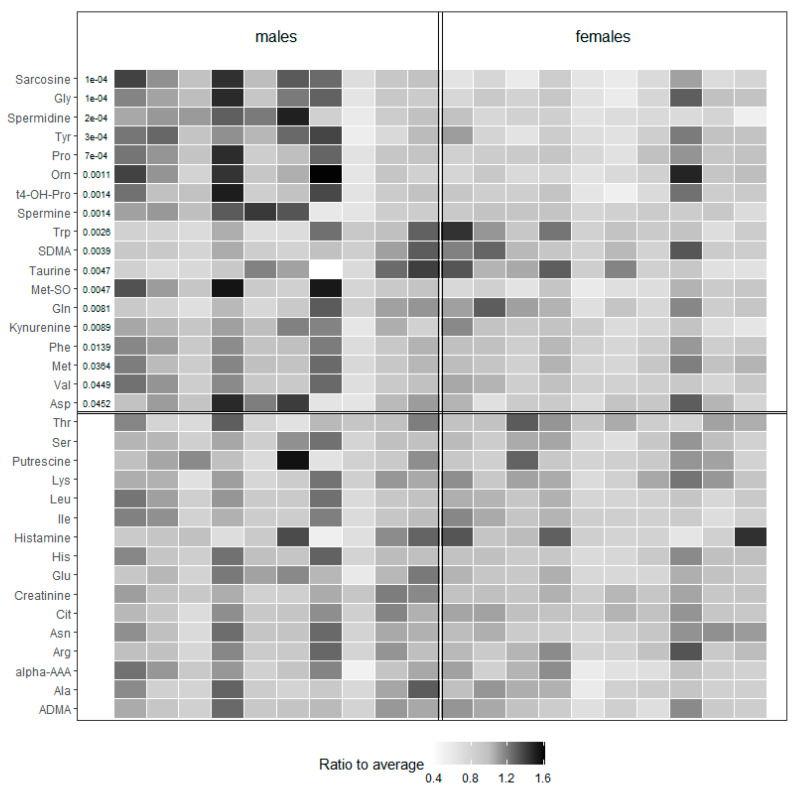
Heat map representing the values of amino acids and biogenic amines of tested male and female animals. Values of each metabolite are color-coded and represent a ratio to average. *p* values indicate the significance and are listed in the beginning of the corresponding line. The lines without *p* values are nonsignificant. Val (Valine), Tyr (Tyrosine), Trp (Tryptophan), Thr (Threonine), t4-OH-Pro (*trans*-4-hydroxyproline), Ser (Serine), SDMA (Symmetric dimethylarginine), Pro (Proline), Phe (Phenylalanine), Orn (Ornithine), Met-So (Methionine-Sulfoxide), Met (Methionine), Lys (Lysine), Leu (Leucine), Ile (Isoleucine), His (Histidine), Gly (Glycine), Glu (Glutamate), Gln (Glutamine), Cit (Citrulline), Asp (Aspartate), Asn (Asparagine), Arg (Arginine), Alpha-AAA (alpha-aminoadipic acid), Ala (Alanine), and ADMA (Asymmetric dimethylarginine).

In the group of amino acids and biogenic amines (Figure 2), up to 45.2% (19/42) of the metabolites was found to be markedly influenced by sex. Of these, 21% were significantly increased (Tryptophan-Trp, Glutamine-Gln, Symmetric dimethylarginine-SDMA, and Taurine) and 79% decreased in females (Glycine-Gly, Tyrosine-Tyr, Proline-Pro, Ornithine-Orn, Phenylalanine-Phe, Methionine-Met, Valine-Val, Aspartate-Asp, Sarcosine, Spermidine and Spermine, *Trans*-4-hydroxyproline-t4-OH-Pro, Methionine-sulfoxide - Met-So, Kynurenine, and *Cis*-4-hydroxyproline-c4-OH-Pro) when compared with males (for concrete values of all metabolites see Supplementary Table S1).

**Figure 3:**
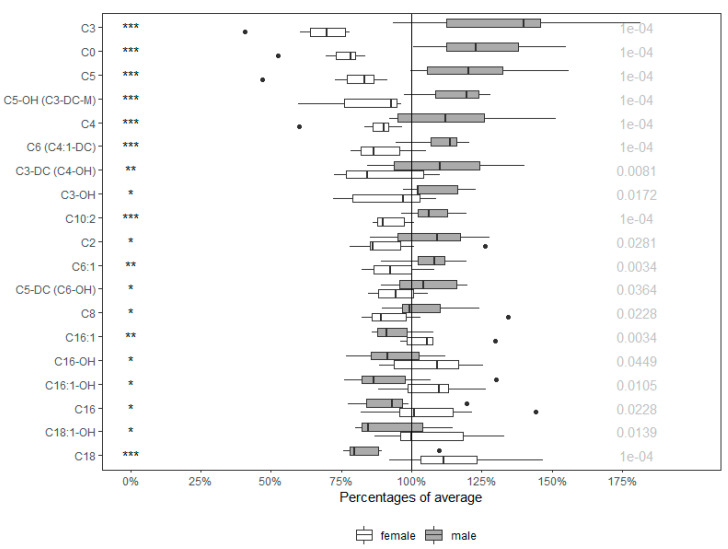
Percentages of average in acylcarnitines of males and females. Values in boxplots are expressed as ratio of each value in respect to the average. Significance is given by * *p* < 0.05, ** *p* < 0.01, and *** *p* < 0.001, respectively, with concrete *p* value at the end of the line. Orn (Ornithine), Tyr (Tyrosine), Met-So (Methionine-Sulfoxide), Gly (Glycine), Pro (Proline), t4-OH-Pro (*trans*-4-hydroxyproline), Asp (Aspartate), Phe (Phenylalanine), Met (Methionine), Val (Valine), Gln (Glutamine), SDMA (Symmetric dimethylarginine), Trp (Tryptophan).

Acylcarnitines, as the main markers of energy metabolism, show a significant sex-dependent pattern. The concentrations of up to 19 from the total of 40 acylcarnitines were statistically different (47.5%) when compared to males. As seen in Figure 3, this group of metabolites was elevated predominantly in males (68%).

**Figure 4:**
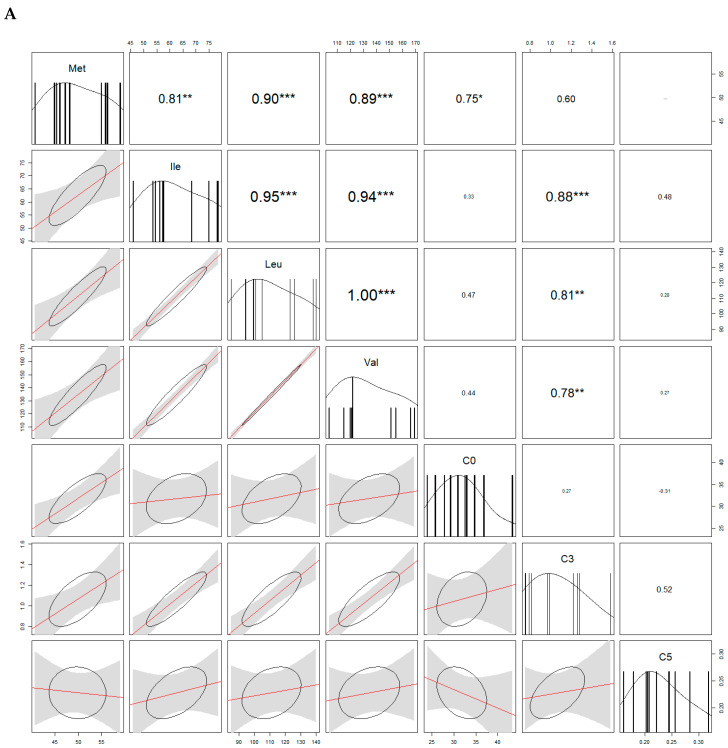

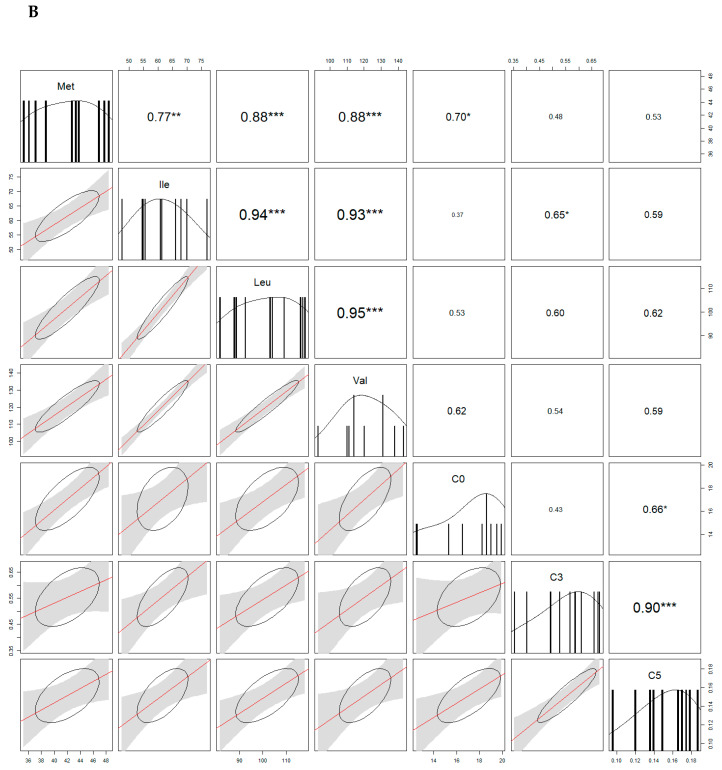
Analysis of correlations (correlograms) among levels of selected carnitines and amino acids in males (**A**) and females (**B**). The coefficients of Spearman Product Moment Correlation are reported. Significance is given by * *p* < 0.05, ** *p* < 0.01, and *** *p* < 0.001, respectively.

Because some acylcarnitines are derived from amino acids, we looked for the correlations. In both sexes, free carnitine (C0) and Met were significantly correlated. We also found positive correlations between propionylcarnitine (C3) and Val/Leu/Ile in males, and between C3 and Ile in females. For more correlations see Figure 4.

Significance analysis of metabolites (SAM) is a well-established statistical method for identification of differentially expressed metabolites in data analysis. It is designed to address the false discovery rate (FDR) when running multiple tests on high-dimensional data. For a variable with scores greater than an adjustable threshold, its relative difference is compared to the distribution estimated by random permutations of the class labels. For each threshold, a certain proportion of the variables in the permutation set will be found to be significant by chance. Table 1 shows the details of these features.

According to univariate analysis, a preliminary overview of potentially significant features of discriminating the conditions was performed. For paired fold change analysis, the algorithm first counts the total number of pairs with fold changes that are consistently above/below the specified Fold Change threshold for each variable. The three most significant features identified by *t*-test are listed in Table 2.

**Figure 5:**
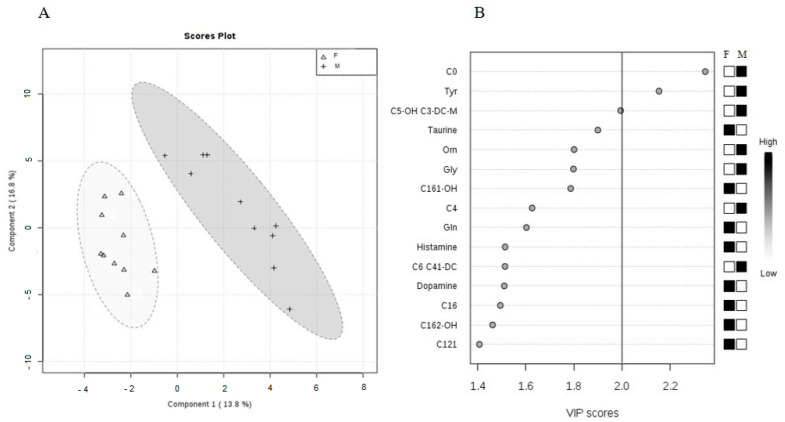
(**A**) Partial least squares-discrimination analysis (PLS-DA) of selected metabolites in male and female animals (PLS-DA scores’ plot includes two components: R2 = 0.93659 and Q2 = 0.44597). In the graphical output, 95% confidence ellipses for specific groups are included. (**B**) Variable importance in projection (VIP) plot, calculated from PLS-DA method, displays the top 15 most important metabolite features identified by PLS-DA. White and black boxes on right indicate relative concentration of corresponding metabolite in blood in descending order of importance. VIP is a weighted sum of squares of the PLS-DA loadings considering the amount of explained Y-variable in each dimension. The most important features have the VIP values of >2.0.

As seen on separate cluster analysis (Figure 5A), the data clearly grouped a set of blood metabolites into females and males. Variable importance in projection (VIP) plot (Figure 5B) showed that C0, Tyr, and partially C5-OH are the most important metabolites identified by Partial least square-discrimination analysis (PLS-DA) with the VIP values of >2.0.

## 3. Discussion

In our study, using a metabolomics approach and data mining analyses, the intersexual differences in the energy metabolism of Wistar rats were revealed. The most important features calculated in the PLS-DA model, according to VIP score, were C0, Tyr, and C5-OH significantly elevated in males.

Basic genetic and physiological differences together with environmental factors result in behavioral and cognitive differences between males and females. Sex differences in the brain and/or in sex-typed behavior and sexual identity should be studied at all points in the life span especially because there are important relationships between sexes and the occurrence, prevalence, age of onset, symptoms, and severity of some diseases [20].

Laboratory rats are an inevitable part of current biomedical research. They are recognized as the preeminent model in numerous fields, including neurobehavioral studies, cancer, and toxicology [12]. Rodent animal models also provide a preclinical platform for characterizing cellular responses to investigational compounds through toxicogenomics analyses of high-throughput molecular data sets [21]. While sex differences influence the application of experimental results into clinical praxis, there is a need to define them also at a preclinical level.

In our study, amino acids and biogenic amines, as well as acylcarnitines, showed sex-specific pattern. Aromatic amino acids (Trp, Tyr, Phe) are the biosynthetic precursors for the neurotransmitters serotonin, dopamine, and norepinephrine. While aromatic amino acids, such as Tyr and Phe, were significantly elevated in the blood plasma of males, Trp was found in higher levels in the blood plasma of females. As shown previously, there is a similarity between rats and humans: The levels of Phe were found to be elevated also in cardiac ventricles of male Sprague–Dawley rats and, more importantly, in human serum of males [19,22]. On the contrary to our data, Krumsiek et al. found higher levels of Trp in males, not in females [22]. In the study of Ruoppolo, Tyr did not display any significant sex differences and Trp was not studied at all [19]. In addition, they found significant sex-related changes in urea cycle [19]. Except SDMA levels (formed by methylation of l-Arginine-l-Arg), Orn and Asp, as well as sarcosine, spermidine, and spermine, were markedly higher in the blood plasma of male rats. These results are consistent with previously published data from a large human population-based study, showing elevated concentrations of Orn, Arg, Gly, and Serine in the blood of males [23]. From the group of aromatic amino acids of our study, Tyr has been identified as the most important feature elevated in males when comparing to females in various analyses. Tyr has been found to enhance neurotransmitter synthesis in active neurons and, thus, may reverse the neurotransmitter depletion and benefit brain function [24]. Significantly elevated levels of amino acids, such as Phe or Tyr, have been associated with the increased risk of hyperglycemia [25] or diabetes mellitus type 2 [25,26]. This indicates that probably this is the reason why diabetes mellitus type 2 is more frequently diagnosed at a lower age and body mass index in men; however, the most prominent risk factor, obesity, is more common in women [26]. Moreover, there is a strong correlation between circulating branched-chain amino acids (BCAAs) and diabetes prediction [27]. BCAAs (Leu, Ile, Val) may influence brain protein synthesis and production of energy and may influence the synthesis of different neurotransmitters. In our study, the levels of Val in males were significantly elevated. Concomitantly, Ruoppolo et al. revealed elevated concentrations of Val and Xle (Ile + Leu) in cardiac ventricles of male Sprague–Dawley rats as well as significantly higher concentrations of Val, Leu, Ile and their first-step degradation products [19].

Acylcarnitines are derived from the oxidation of a variety of metabolic fuels, including β-oxidation of fatty acids (FAs). Acylcarnitines are formed to facilitate the transport of long-chain fatty acids into mitochondria. They are also formed by equilibration of mitochondrial acyl Coenzyme A with their cognate acylcarnitines through the action of carnitine acyltransferase enzymes [28]. What is important is that some carnitines are dependent on branched-chain amino acids. We found the positive correlation between Met and CO carnitine in blood plasma of males and females and positive correlations between Val and C3, Ile and C3, and Leu and C3 in males, and Ile and C3 in females. Generally, in our study, most carnitines were significantly elevated in male rats because of the higher rate of energy metabolism described previously [29,30]. One of the reasons why males have a higher energy metabolism is that they have a larger mass of organs and more muscles, therefore, higher energy requirements, which are associated with a higher oxidation of fatty acids [31]. However, our data show some discrepancies when comparing with others. In previous studies, C6, C10:2, and C8 were significantly higher in the hearts of male Sprague-Dawley rats [19]. At cardiac levels, also C2, C10, C12, C6-DC, C14:2, C14:1, C14, C8-DC, C16, C10-DC, C16-OH, C18, C18:1-OH, C6:1, C10:2, C12-OH, C14-OH, C16:1-OH, and C18:2 were significantly higher in males than in females. Only C3-DC, C4-DC, and C4-OH were higher in female hearts than in male ones [19]. Surprisingly, we found higher levels of long-chain carnitines C16, C18 and C16-OH, C16:1, C16:1-OH, and C18:1-OH in the blood of females. However, C16, C16-OH, and C16:1-OH, as well as C18, have been previously found in elevated concentrations in male rats and humans [19,23]. We further found two acylcarnitines to be described as the most important among others and to be significantly elevated in males: C0 and C5-OH. C0, or free carnitine, is a vitamin-like compound that plays important roles in fatty acid β-oxidation and the control of the mitochondrial coenzyme A/acetyl-CoA ratio [32]. Carnitine levels differ by sex and age [33]. It has been previously shown that the plasma-free carnitine (C0) concentration is significantly higher in males than in females [34]. Thus, C0 could be described as a sex-specific feature. C5-OH (hydroxyvalerylcarnitine) is a derivate of carnitine and is classified as a member of FAs. It was described as a dynamic biomarker candidate for physical activity [35]. However, there is a lack of information about this acylcarnitine and its potential sex specificity.

## 4. Materials and Methods

### 4.1. Experimental Design

In the experiment, 10 adult (sexually mature) females and 10 adult (sexually mature) males of Wistar rats (Dobrá Voda, Slovak) aged 60 days were used. Animals were kept under standard conditions with a room temperature of 21–24 °C, relative humidity of 50–65%, and a 12:12 h regimen. Animals were fed with a standard rat pelleted diet (Peter Miško, Snina, Slovakia) ad libitum according to EU animal feed legislation and guidance and water was freely available. The animals were weighed and food intake was monitored twice during the experiment. To mimic human conditions, sex differences of selected amino acids and acylcarnitines were monitored independently on the stage of the reproductive cycle. The female rats were born on the same day and we assume that they were synchronized. From previous studies, we know that female rats become sexually mature at about the sixth week [12]. The animals were handled by the guidelines established by Law No. 377 and 436/2012 of Slovak Republic for the Care and Use of Laboratory Animals (Ro-2866/16-221).

### 4.2. Blood Collection and Metabolomics’ Measurement

The blood from all experimental animals was collected at one session (10:00 a.m.) from the great saphenous vein (*vena saphena magna*) in a total volume of 100 µL into microtubes with heparin. The place of the collection was preshaved and treated with a disinfectant. After isolating, blood plasma was stored at −80 °C. Frozen plasma was thawed on ice and centrifuged and the supernatant was used for further analysis. The samples were measured by the AbsoluteIDQ p180 kit in the laboratory of BIOCRATES Life Sciences AG in Innsbruck (Austria). Flow injection analysis (FIA-MS/MS) and liquid chromatography-tandem mass spectrometry-based (LC-MS/MS) targeted-metabolomics’ measurement of a selected groups of amino acids, biogenic amines, and acylcarnitines (as the representatives of energy metabolism) was performed on plasma samples (for individual metabolites see Supplementary Table S2). Concrete, for the analysis of acylcarnitines, samples were analyzed using a FIA-MS/MS. Sample analyses of amino acids and biogenic amines were performed by a UPLC (ultra-high-pressure liquid chromatography) tandem MS method using a reversed phase analytical column for analyte separation (LC-MS/MS). The fully automated assay was based on PITC (phenylysothiocyanate) derivatization in the presence of internal standards followed by FIA-MS/MS and LC-MS/MS using a SCIEX 4000 QTRAP^®^ (SCIEX, Darmstadt, Germany) or a Waters XEVO™ TQMS (Waters, Vienna, Austria) instrument with electrospray ionization. The assay was based on the principle described in the study of [36]. Determined values were log2-transformed to obtain normally distributed data and to stabilize the variance.

### 4.3. Statistical Analysis

Quantification of metabolite concentrations and quality assessment was performed using the MetIQ software package (BIOCRATES Life Sciences AG, Innsbruck, Austria). Internal standards served as the reference for the metabolite concentration calculations. Univariate (*t*-test) and multivariate statistics (partial least squares-discrimination analysis PLS-DA) as well as the variable importance in projection (VIP) plot, were performed using MetaboAnalyst 3.0 (Xia Lab @ McGill, Ste. Anne de Bellevue, Quebec, US) [37]. Cross-validation of PLS-DA classification applied five components for selecting optimal number of components. The Leave-one-out cross-validation (LOOCV) method was used. The performance of measures was considered as Accuracy, coefficient of determination (R2-R squared), and Quartile squared (Q2). As a part of the PLS-DA method, a variance importance in projection (VIP) score was measured. VIP score is a measure of a feature’s importance in the PLS-DA model. It summarizes the contribution a feature makes to the model. The VIP score of a feature is calculated as a weighted sum of the PLS weight. PLS weight is the squared correlations between the PLS-DA components and the original feature. Tables, heat map, and box plots were performed using GraphPad 6.0 (GraphPad Software, Inc., San Diego, CA, USA) and programming language R (version 3.6.0) with standard library and libraries ggplot2 (version 3.1.1), ggpubr (version 0.2), psych (version 1.8.12), and GGally (version 1.4.0).

## 5. Conclusions

Complex metabolic processes represent an important understanding of biological body functioning. Because laboratory rats represent a key preclinical step to clinical investigations, there is a need to define the similarities and differences between rats and humans. In order to achieve an individual therapeutic approach, it is essential to know the fundamental differences between male and female metabolism. In our study, we revealed C0, Tyr, and C5-OH as the most important features elevated in males. It is obvious that many differences emerge, in particular, in energy metabolism related to sex, and that metabolomics along with other –omics’ technologies can help to dissect sex-related traits in pathophysiology. Our study provides an important insight into preclinical sex-specific differences of metabolism and may help in the future in clinical diagnostics of many diseases. It is of note that the metabolomic status of individuals may be dependent not only on sexual differences but also on age and, thus, the sex-dependent characteristics of individual metabolites seen in this study might vary with different age of rats. Moreover, the results might differ with the strains of rats. Therefore, further studies are necessary to reveal these differences.

## Figures and Tables

**Table 1:** Important features identified by significance analysis of metabolites (SAM): Butyrylcarnitine/Isobutyrylcarnitine (C4), carnitine (free) (C0), glycine (Gly), hexanoylcarnitine (C6) (C4:1-DC), hydroxyvalerylcarnitine (C5-OH) (C3-DC-M), ornithine (Orn), tyrosine (Tyr).

Compound	d Value	SD	Raw p	q Value
C0	5.5527	0.27896	0	0
Tyr	4.0661	0.33172	0.00025	0.0059235
C5-OH (C3-DC-M)	3.8686	0.33952	0.000625	0.0098725
Orn	3.4304	0.35729	0.001375	0.014216
Gly	3.3856	0.35913	0.0015	0.014216
C4	2.6289	0.39057	0.01525	0.099959
C6 (C4:1-DC)	2.5906	0.39214	0.016875	0.099959

**Table 2:** Important features identified by *t*-test: Carnitine (free) (C0), hydroxyvalerylcarnitine (C5-OH) (C3-DC-M), false discovery rate (FDR), tyrosine (Tyr).

Compound	*t* Stat	*p* Value	−log10(p)	FDR
C0	−5.5527	0.000028526	4.5448	0.0022821
Tyr	−4.0661	0.00072486	3.1397	0.028994
C5-OH (C3-DC-M)	−3.8686	0.0011255	2.9486	0.030014

## Supplementary Materials

The following are available online, Table S1: Values of metabolites analyzed in the experiment; Table S2: Abbreviations of metabolites analyzed.

## Abbreviations

Arg	arginine
Asp	aspartate
BCAAs	branched-chain amino acids
C0	carnitine (free)
C2	acetylcarnitine
C3	propionylcarnitine
C3-DC	malonylcarnitine
C4-DC	methylmalonylcarnitine
C4-OH	hydroxybutyrylcarnitine
C4-OH-Pro	*cis*-4-hydroxyproline
C5-OH	hydroxyvalerylcarnitine
C6	hexanoylcarnitine
C6-DC	methylglutarylcarnitine
C6:1	hexenoylcarnitine
C8	octanoylcarnitine
C8-DC	suberylcarnitine
C10	decanoylcarnitine
C10:2	decadienoylcarnitine
C10-DC	decanedioylcarnitine
C12	dodecanoylcarnitine
C12-OH	3-hydroxydodecanoylcarnitine
C14	myristylcarnitine
C14:1	myristoleylcarnitine
C14:2	tetradecadienoylcarnitine
C14-OH	3-hydroxytetradecanoylcarnitine
C16	palmitoylcarnitine
C16:1	palmitoleylcarnitine
C16-OH	hydroxypalmitoylcarnitine
C16:1-OH	hydroxypalmitoleylcarnitine
C18	stearylcarnitine
C18:2	linoleylcarnitine
C18:1-OH	hydroxyoleylcarnitine
FAs	fatty acids
FDR	false discovery rate
Gln	glutamine
Gly	glycine
Ile	isoleucine
Leu	leucine
Met	methionine
Met-So	methionine-sulfoxide
Orn	ornithine
Phe	phenylalanine
Pro	proline
SAM	significance analysis of metabolites
SDMA	symmetric dimethylarginine
Ser	serine
Trp	tryptophan
Tyr	tyrosine
t4-OH-Pro	*trans*-4-hydroxyproline
Val	valine
VIP	variance importance in projection
