# Supplementary material for: Sexual Dimorphism in Energy Metabolism of Wistar Rats Using Data Analysis
Source: Molecules. 2020 May 18;25(10):2353. doi: 10.3390/molecules25102353 (PMC7287681; doi:10.3390/molecules25102353)
Supplement: Supplementary file 1 [file molecules-25-02353-s001.pdf]

molecules

MDPI

Article

Sexual Dimorphism in Energy Metabolism of Wistar Rats Using Data Analysis

Andrea Leskanicova [1], Olga Chovancova [2], Marian Babincak [1], Ludmila Verboova [3], Zuzana Benetinova [3], Denisa Macekova [2], Jozef Kostolny [2], Benadik Smajda [1,*] and Terezia Kiskova [1,*]

[1] Institute of Biology and Ecology, Faculty of Sciences, University of Pavol Jozef Šafárik in Košice, Šrobárova 2, 041 80 Košice, Slovak Republic; andrea.stafurikova@student.upjs.sk (A.L.); marian.babincak@student.upjs.sk (M.B.)
[2] Department of Informatics, Faculty of Management Sciences and Informatics, University of Žilina, Univerzitná 8215/1, 010 26 Žilina, Slovak Republic; olga.chovancova@fri.uniza.sk (O.C.); denisa.macekova@fri.uniza.sk (D.M.); jozef.kostolny@fri.uniza.sk (J.K.)
[3] Department of Pathology, Faculty of Medicine, University of Pavol Jozef Šafárik in Košice, Rastislavova 43, 040 01 Košice, Slovak Republic; ludmila.verboova@upjs.sk (L.V.); zuzana.benetinova@upjs.sk (Z.B.)
* Correspondence: benadik.smajda@upjs.sk (B.S.); terezia.kiskova@upjs.sk (T.K.); Tel.: +421-55-234-1216 (T.K.); Fax: +421-55-622-2124 (T.K.)

Table 1. Values of metabolites analyzed in the experiment.

Metabolite (μM)	Female	Male
**C0**	**17.69 ± 2.431 *****	29.86 ± 4.475
**C2**	**11.37 ± 1.894 ***	13.01 ± 1.828
**C3**	**0.5836 ± 0.1106 *****	01.159 ± 0.2450
**C3-DC (C4-OH)**	**0.06929 ± 0.01176 ****	0.08489 ± 0.01522
**C3-OH**	**0.008514 ± 0.001295 ***	0.01006 ± 0.0009112
**C3:1**	0.009329 ± 0.002053	0.0102 ± 0.001154
**C4**	**0.3979 ± 0.05378 *****	0.5263 ± 0.09839
**C4:1**	0.01487 ± 0.002644	0.01526 ± 0.001764
**C5**	**0.1598 ± 0.02957 *****	0.2480 ± 0.04029
**C5-DC (C6-OH)**	**0.01073 ± 0.0008730 ***	0.01193 ± 0.001342
**C5-M-DC**	0.02451 ± 0.003700	0.02377 ± 0.001730
**C5-OH (C3-DC-M)**	**0.03326 ± 0.005317 *****	0.04561 ± 0.004371
**C5:1**	0.02036 ± 0.001833	0.02173 ± 0.002696
**C5:1-DC**	0.009457 ± 0.0009395	0.009043 ± 0.001102
**C6 (C4:1-DC)**	**0.05706 ± 0.006587 *****	0.07064 ± 0.005369
**C6:1**	**0.02036 ± 0.002069 ****	0.02313 ± 0.002057
**C7-DC**	0.008143 ± 0.0008751	0.009357 ± 0.001504
**C8**	**0.06494 ± 0.01176 ***	0.07001 ± 0.007655
**C9**	0.01111 ± 0.0005960	0.01206 ± 0.001775
**C10**	0.03496 ± 0.004365	0.03653 ± 0.003538
**C10:1**	0.05257 ± 0.005530	0.05304 ± 0.01013
**C10:2**	**0.01919 ± 0.001176 *****	0.02229 ± 0.001755
**C12**	0.03193 ± 0.002690	0.03333 ± 0.003771
**C12-DC**	0.04391 ± 0.001279	0.04431 ± 0.002102
**C12:1**	0.05577 ± 0.006487	0.04981 ± 0.01018
**C14**	0.04501 ± 0.006896	0.03999 ± 0.004532
**C14:1**	0.05233 ± 0.005825	0.04761 ± 0.006719

**C14:1-OH**	0.009529 ± 0.001705	0.008771 ± 0.0009903
**C14:2**	0.008757 ± 0.001468	0.008486 ± 0.0005586
**C14:2-OH**	0.007471 ± 0.0008241	0.0068 ± 0.001088
**C16**	**0.09847 ± 0.01814 ***	0.08574 ± 0.01240
**C16-OH**	**0.0072 ± 0.0009735 ***	0.006357 ± 0.0008644
**C16:1**	**0.03349 ± 0.003485 ****	0.02961 ± 0.002455
**C16:1-OH**	**0.01149 ± 0.001318 ***	0.01003 ± 0.001961
**C16:2**	0.005786 ± 0.0009404	0.005657 ± 0.0006321
**C16:2-OH**	0.01043 ± 0.001390	0.01026 ± 0.001503
**C18**	**0.07547 ± 0.01215 *****	0.05616 ± 0.007502
**C18:1**	0.1095 ± 0.02569	0.09933 ± 0.01318
**C18:1-OH**	**0.01391 ± 0.002164 ***	0.0121 ± 0.001870
**C18:2**	0.02187 ± 0.004628	0.02231 ± 0.003252

**Ala**	465.7 ± 71.47	421.1 ± 82.94
**Arg**	157.7 ± 19.25	156.4 ± 26.00
**Asn**	28.47 ± 3.061	30.80 ± 5.535
**Asp**	**12.83 ± 2.478 ***	15.94 ± 4.050
**Cit**	73.44 ± 7.500	69.59 ± 10.53
**Gln**	**667.4 ± 81.54 ****	561.6 ± 122.3
**Glu**	81.64 ± 15.76	87.64 ± 11.56
**Gly**	**519.0 ± 61.26 *****	728.9 ± 111.2
**His**	53.63 ± 4.078	60.76 ± 9.361
**Ile**	64.37 ± 8.848	66.70 ± 10.50
**Leu**	107.0 ± 12.22	117.2 ± 18.41
**Lys**	481.4 ± 50.77	448.1 ± 89.71
**Met**	**43.34 ± 4.708 ***	49.86 ± 6.507
**Orn**	**42.39 ± 5.499 ****	63.20 ± 15.23
**Phe**	**45.04 ± 5.490 ***	52.04 ± 5.503
**Pro**	**175.7 ± 20.04 *****	228.7 ± 41.23
**Ser**	337.3 ± 35.36	358.9 ± 48.96
**Thr**	230.4 ± 29.50	198.4 ± 50.15
**Trp**	**80.53 ± 13.92 ****	61.67 ± 14.09
**Tyr**	**50.24 ± 11.59 *****	72.66 ± 8.965
**Val**	**124.3 ± 16.32 ***	142.4 ± 22.21

**Ac-Orn**	0.02914 ± 0.04828	0.05171 ± 0.06201
**ADMA**	0.5843 ± 0.07151	0.5789 ± 0.07936
**alpha-AAA**	0.9631 ± 0.2185	1.067 ± 0.1510
**c4-OH-Pro**	**0.1077 ± 0.07170 ***	0.1477 ± 0.06590
**Carnosine**	0.1889 ± 0.06601	0.1854 ± 0.1144
**Creatinine**	17.11 ± 1.754	15.94 ± 1.803
**DOPA**	0.04057 ± 0.01726	0.04814 ± 0.002742
**Dopamine**	0.1006 ± 0.006223	0.07414 ± 0.04883
**Histamine**	0.6670 ± 0.1389	0.5460 ± 0.1507
**Kynurenine**	**2.363 ± 0.4258 ****	2.736 ± 0.2339
**Met-SO**	**3.820 ± 0.4884 ****	5.391 ± 1.364
**Putrescine**	0.5493 ± 0.09430	0.5763 ± 0.1630
**Sarcosine**	**5.500 ± 0.9958 *****	8.637 ± 1.281
**SDMA**	**0.2100 ± 0.03215 ****	0.1729 ± 0.01706
**Serotonin**	0.04986 ± 0.03872	0.04929 ± 0.03820
**Spermidine**	**2.159 ± 0.3407 *****	3.021 ± 0.5259

**Spermine**	**0.4237 ± 0.05173 ****	0.5463 ± 0.1290
**t4-OH-Pro**	**78.29 ± 9.469 ****	101.7 ± 21.77
**Taurine**	**177.1 ± 34.34 ****	127.6 ± 39.33

Data are expressed as mean ± SD. Significance versus male is by *P < 0.05; **P < 0.01 and ***P < 0.001, respectively.

Table 2. Abbreviations of metabolites analyzed.

BC code	Analyte
**Amino acids**	
Ala	Alanine
Arg	Arginine
Asn	Asparagine
Asp	Aspartate
Cit	Citrulline
Gln	Glutamine
Glu	Glutamate
Gly	Glycine
His	Histidine
Ile	Isoleucine
Leu	Leucine
Lys	Lysine
Met	Methionine
Orn	Ornithine
Phe	Phenylalanine
Pro	Proline
Ser	Serine
Thr	Threonine
Trp	Tryptophan
Tyr	Tyrosine
Val	Valine
**Biogenic Amines**	
Ac-Orn	Acetylornithine
ADMA	Asymmetric dimethylarginine
SDMA	Symmetric dimethylarginine
alpha-AAA	alpha-Aminoadipic acid
Histamine	Histamine
Met-SO	Methionine-Sulfoxide
Kyn	Kynurenine
Putrescine	Putrescine
Sarcosine	Sarcosine
Spermidine	Spermidine
Spermine	Spermine
Serotonin	Serotonin
PEA	Phenylethylamine
Nitro-Tyr	Nitrotyrosine
c4-OH-Pro	cis-4-Hydroxyproline
t4-OH-Pro	trans-4-Hydroxyproline
Creatinine	Creatinine
Carnosine	Carnosine
Taurine	Taurine
DOPA	Dihydroxyphenylalanine

Dopamin	Dopamin
**Acylcarnitine**	
C0	Carnitine (free)
C2	Acetylcarnitine
C3	Propionylcarnitine
C3:1	Propenoylcarnitine
C3-OH	Hydroxypropionylcarnitine
C4	Butyrylcarnitine / Isobutyrylcarnitine
C4:1	Butenoylcarnitine
C4-OH (C3-DC)	Hydroxybutyrylcarnitine (Malonylcarnitine)
C5	Isovalerylcarnitine / 2-Methylbutyrylcarnitine / Valerylcarnitine
C5:1	Tiglylcarnitine / 3-Methyl-crotonylcarnitine
C5:1-DC	Glutaconylcarnitine / Mesaconylcarnitine
C5-DC (C6-OH)	Glutarylcarnitine (Hydroxyhexanoylcarnitine [= Hydroxycaproylcarnitine])
C5-M-DC	Methylglutarylcarnitine
C5-OH (C3-DC-M)	Hydroxyisovalerylcarnitine / Hydroxy-2-methylbutyryl /Hydroxyvalerylcarnitine (Methylmalonylcarnitine)
C6 (C4:1-DC)	Hexanoylcarnitine [= Caproylcarnitine] (Fumarylcarnitine)
C6:1	Hexenoylcarnitine
C7-DC	Pimelylcarnitine
C8	Octanoylcarnitine [= Caprylylcarnitine]
C9	Nonanoylcarnitine [= Pelargonylcarnitine]
C10	Decanoylcarnitine [= Caprylcarnitine]
C10:1	Decenoylcarnitine
C10:2	Decadienoylcarnitine
C12	Dodecanoylcarnitine [= Laurylcarnitine]
C12:1	Dodecenoylcarnitine
C12-DC	Dodecanedioylcarnitine
C14	Tetradecanoylcarnitine [= Myristylcarnitine]
C14:1	Tetradecenoylcarnitine [= Myristoleylcarnitine]
C14:1-OH	Hydroxytetradecenoylcarnitine [= Hydroxymyristoleylcarnitine]
C14:2	Tetradecadienoylcarnitine
C14:2-OH	Hydroxytetradecadienoylcarnitine
C16	Hexadecanoylcarnitine [= Palmitoylcarnitine]
C16:1	Hexadecenoylcarnitine [= Palmitoleylcarnitine]
C16:1-OH	Hydroxyhexadecenoylcarnitine [= Hydroxypalmitoleylcarnitine]
C16:2	Hexadecadienoylcarnitine
C16:2-OH	Hydroxyhexadecadienoylcarnitine
C16-OH	Hydroxyhexadecanolycarnitine [= Hydroxypalmitoylcarnitine]
C18	Octadecanoylcarnitine [= Stearylcarnitine]
C18:1	Octadecenoylcarnitine [= Oleylcarnitine]
C18:1-OH	Hydroxyoctadecenoylcarnitine [= Hydroxyoleylcarnitine]
C18:2	Octadecadienoylcarnitine [= Linoleylcarnitine]
